# Widespread Cortical Thickness Is Associated With Neuroactive Steroid Levels

**DOI:** 10.3389/fnins.2019.01118

**Published:** 2019-11-12

**Authors:** Rajendra A. Morey, Sarah L. Davis, Courtney C. Haswell, Jennifer C. Naylor, Jason D. Kilts, Steven T. Szabo, Larry J. Shampine, Gillian J. Parke, Delin Sun, Chelsea A. Swanson, Henry R. Wagner, Mid-Atlantic MIRECC Workgroup, Christine E. Marx

**Affiliations:** ^1^Mid-Atlantic Mental Illness Research, Education and Clinical Center, Durham VA Medical Center, U.S. Department of Veteran Affairs, Durham, NC, United States; ^2^Department of Psychiatry and Behavioral Sciences, Duke University Medical Center, Durham, NC, United States; ^3^Brain Imaging and Analysis Center, Duke University, Durham, NC, United States

**Keywords:** neuroactive steroids, cortical thickness, MRI, neuroregeneration, neuroprotection, gray matter

## Abstract

**Background:**

Neuroactive steroids are endogenous molecules with regenerative and neuroprotective actions. Both cortical thickness and many neuroactive steroid levels decline with age and are decreased in several neuropsychiatric disorders. However, a systematic examination of the relationship between serum neuroactive steroid levels and *in vivo* measures of cortical thickness in humans is lacking.

**Methods:**

Peripheral serum levels of seven neuroactive steroids were assayed in United States military veterans. All (*n* = 143) subsequently underwent high-resolution structural MRI, followed by parcellelation of the cortical surface into 148 anatomically defined regions. Regression modeling was applied to test the association between neuroactive steroid levels and hemispheric total gray matter volume as well as region-specific cortical thickness. False discovery rate (FDR) correction was used to control for Type 1 error from multiple testing.

**Results:**

Neuroactive steroid levels of allopregnanolone and pregnenolone were positively correlated with gray matter thickness in multiple regions of cingulate, parietal, and occipital association cortices (*r* = 0.20–0.47; *p* < 0.05; FDR-corrected).

**Conclusion:**

Positive associations between serum neuroactive steroid levels and gray matter cortical thickness are found in multiple brain regions. If these results are confirmed, neuroactive steroid levels and cortical thickness may help in monitoring the clinical response in future intervention studies of neuroregenerative therapies.

## Introduction

Neuroactive steroids are endogenous molecules that are enriched in human brain, where they are synthesized *de novo* from cholesterol. They are also produced in the adrenal glands and other peripheral tissues. A number of neuroactive steroids are neuroactive, exhibiting rapid actions at inhibitory GABA_A_ receptors, excitatory NMDA receptors, and other ligand-gated ion channel receptors (Paul and Purdy, 1992). Importantly, a number of neuroactive steroids exhibit neuroregenerative, neuroprotective, and neurotrophic actions, in addition to anti-inflammatory effects. Furthermore, accruing evidence suggests that neuroactive steroids may be effective treatment interventions for several CNS disorders where cortical thickness is reduced and for which neuroactive steroid interventions show potential (Irwin et al., 2014; Vallée, 2016). Neuroactive steroids are thus plausible modulators of brain structure and function, but investigation of neuroactive steroids in conjunction with magnetic resonance imaging (MRI) in humans is currently scant.

The neuroprotective and neuroregenerative potential of neuroactive steroids is particularly promising for developing new therapeutics utilizing neuroactive steroids as interventions and neuroimaging to assess potential treatment effects. For instance, allopregnanolone reduces oxidative stress (Qian et al., 2015) and exhibits neuroprotective actions in animal models of stroke and traumatic brain injury (TBI) (Djebaili et al., 2005; Sayeed et al., 2006), decreases cytokine expression following TBI (He et al., 2004), reduces microglia activation (Chen et al., 2011), and enhances myelin basic protein expression (Ghoumari et al., 2003). Pregnenolone protects against glutamate and amyloid beta protein (Gursoy et al., 2001), while DHEAS and DHEA are protective against excitotoxicity (Kimonides et al., 1998), decrease apoptosis (Charalampopoulos et al., 2004), and enhance axonal and dendritic outgrowth (Compagnone and Mellon, 1998). Allopregnanolone is a GABAergic neuroactive steroid that dose-dependently increases proliferation of rodent and human neural progenitor cells, restores neurogenesis and reverses learning and memory deficits in a mouse model of Alzheimer’s disease (Wang et al., 2010), promotes neurogenesis and survival of newly generated neural cells, and is capable of restoring hippocampal-dependent cognitive performance (Chen et al., 2011; Singh et al., 2012). Consistent with a role for allopregnanolone in the neurobiology and therapeutics of degenerative disorders, allopregnanolone levels are decreased in post-mortem brain tissue of patients with Alzheimer’s disease, and reductions are associated with the presence of the ApoE4 allele (Marx et al., 2006b; Naylor et al., 2010). Peripheral levels of some neuroactive steroids decline with age (Schumacher et al., 2003), and appear to be altered in PTSD (Rasmusson et al., 2006, 2017), schizophrenia (Marx et al., 2009), bipolar disorder (Youssef et al., 2015), depression (Uzunova et al., 1998), and TBI (Marx et al., 2016). Like allopregnanolone, DHEA (Suzuki et al., 2004) and pregnenolone sulfate (Mayo et al., 2005; Xu et al., 2012) also appear to enhance neurogenesis in rodent models.

Few neuroimaging studies in humans have examined associations between neuroactive steroids and brain structure or function despite clear evidence of the regenerative, neuroprotective, neurotrophic, and anti-inflammatory actions of neuroactive steroids in rodents. Many neuroactive steroids in their unsulfated forms are lipophilic and cross the blood-brain barrier. Therefore, closely correlated levels of neuroactive steroids in peripheral blood, cerebrospinal fluid (CSF), and brain would be hypothesized, and there is evidence to support a CSF-brain correlation of DHEA and pregnenolone in human post-mortem brains (Naylor et al., 2008) and a brain-blood correlation of pregnenolone in rats (Marx et al., 2006a). Prior structural MRI investigations have found cortical thickness is associated with DHEA levels during development (Nguyen et al., 2013). Aging is accompanied by cortical thinning throughout adulthood, which appears to be a result of cellular shrinkage and reduced dendritic arborization, rather than neuronal death (Morrison and Hof, 1997). Cortical thinning is also associated with multiple disorders, including first-episode psychosis (Plitman et al., 2016), schizophrenia spectrum symptoms (Watsky et al., 2016), schizophrenia (Rimol et al., 2010; van Erp et al., 2016), bipolar disorder (Hanford et al., 2016), ADHD (Fernández-Jaén et al., 2014), and Alzheimer’s disease (Dickerson et al., 2009), whereas greater cortical thickness appears to be associated with autism (Hyde et al., 2010), and IQ (Narr et al., 2005). Genetic factors also influence cortical thickness, as underscored by its high heritability (*h*^2^ = 0.81) (Panizzon et al., 2009). Interestingly, cortical thickness differs between males and females depending on brain region and age, such that generally some brain regions are thicker in men while other brain regions are thicker in women according to a pattern that varies with age (Mutlu et al., 2013).

Our goal was to evaluate possible associations of neuroactive steroid levels with neuronal integrity, inferred from *in vivo* MRI measurement of cortical gray matter thickness. We quantified seven neuroactive steroids in male military veterans who had undergone MRI scans, followed by parcellelation of the cortical surface. We hypothesized that serum neuroactive steroid levels would be positively correlated with mean cortical thickness of prefrontal and association cortices, given the steroid-induced remodeling that these cortical structures undergo at various developmental periods and the neuroregenerative and neuroprotective properties of these molecules. Finally, we hypothesized that neuroactive steroids may exert stronger influence on cortical thickness for young adults but this influence might be weak in older adults.

## Participants and Methods

### Participants

Male participants were recruited from a repository of United States military veterans (VA Mid-Atlantic MIRECC, Durham, NC, United States) who served in the United States Military since September 11, 2001. All participants provided written informed consent to procedures that were reviewed and approved by the Institutional Review Boards at Duke University and Durham VA Medical Centers. The overall study included *n* = 143 participants and a subsample of *n* = 115 participants, which for convenience are referred to as full-sample (mean age 38.60, SD = 7.14, range 21–65) and subsample (mean age 39.64, SD = 9.78, range 21–65), respectively. Pregnenolone, allopregnanolone, pregnanolone, androsterone, and progesterone levels were only available in a subsample of 115 of the 143 participants. All participants underwent high-resolution structural MRI.

### Participant Screening and Clinical Assessment

The screening process excluded major neurological disorders, contraindication to MRI (e.g., metal implants), history of moderate or severe TBI, substance dependence, and age over 65 years. In addition, all Axis I disorders were excluded with the following exceptions: major depressive disorder, PTSD, past alcohol or substance abuse, and current or past nicotine dependence. Specifically, individuals with current and past alcohol or substance dependence, as well as current alcohol or substance abuse, were excluded from participation. Current alcohol or substance abuse is defined as any use in the past 1 month. Use that occurred prior to the past 1 month, is classified as past alcohol or substance abuse. No toxicology screening was done to verify lack of current substance abuse based on empirical evidence that self-report provides an accurate account (Calhoun et al., 2000). Recruitment of United States Military veterans from recent conflicts meant that excluding diagnoses such as PTSD and major depressive disorder was impractical to obtain a sufficient sample size. Therefore, statistical methods were introduced to account for these conditions. Participants were assessed for lifetime occurrence of psychological trauma [Traumatic Life Events Questionnaire (TLEQ; Kubany et al., 2000)], combat exposure [Combat Exposure Scale (CES; Lund et al., 1984)], alcohol abuse [Alcohol Use Disorders Test (AUDIT; Saunders et al., 1993)], smoking status assessed by the Fagerstrom test for nicotine dependence (Heatherton et al., 1991), and depressive symptoms [Beck Depression Inventory-II (BDI-II; Beck et al., 1996)]. Child trauma was coded from the TLEQ based on the number of categories (0, 1, 2, or more) of trauma experienced. Diagnosis of PTSD was ascertained with the Clinician Administered PTSD Scale (CAPS). Severity of TBI was operationalized based on the criteria for mild TBI that are defined by the American College of Rehabilitation Medicine (ACRM). Individuals who exceeded the ACRM criteria were categorized in the moderate to severe category and therefore excluded.

### MRI Acquisition

All images were acquired on a GE 3-Tesla scanner equipped with an 8-channel head coil at Duke University using high-resolution T1-weighted whole-brain axial images with 1 mm isotropic voxels. High-resolution T1-weighted whole-brain axial images with 1-mm isotropic voxels were obtained with array spatial sensitivity encoding technique (ASSET) and fast spoiled gradient-recall (3D-FSPGR). Image parameters were optimized for contrast between white matter, gray matter, and CSF on either the (1) GE Discovery MR750 (*n* = 162) (TR/TE/flip angle = 7.484-ms/2.984-ms/12°, FOV = 256-mm, 1-mm slice thickness, 166 slices, 256 × 256 matrix, 1 excitation), or the (2) GE EXCITE (*n* = 90) (TR/TE/flip angle = 8.208-ms/3.22-ms/12°, FOV = 256-mm, 1-mm slice thickness, 166 slices, 256 × 256 matrix, 1 excitation).

### Regional Cortical Thickness Analysis

All T1-images were visually inspected to assure sufficient quality for automated segmentation and labeling, which were performed using the FreeSurfer image analysis suite^1^ (version 5.3.0) and its library tool *recon-all*. Details of FreeSurfer parcellations have been previously described (Fischl et al., 2004; Destrieux et al., 2010). Briefly, the technique automatically assigns a neuroanatomical label to each location of a cortical surface model based on probabilistic information estimated from a manually labeled training set and geometric information derived from the cortical model. Mean cortical thickness measures were calculated for 148 neuroanatomical regions (74 per hemisphere) with the aparc.a2009s template (Destrieux et al., 2010).

### Quantification of Serum Neuroactive Steroid Levels

All serum samples were drawn between 10:30 AM and 2:30 PM to minimize the effects of diurnal variations and collected at the time that participants entered the registry. Peripheral serum levels of three neuroactive steroids were quantified via radioimmunoassay: dehydroepiandrosterone (DHEA; full-sample), dehydroepiandrosterone sulfate (DHEAS; full-sample), and progesterone (subsample). Peripheral serum levels of four neuroactive steroids (allopregnanolone, pregnenolone, androsterone, and pregnanolone) were quantified in the subsample by mass spectrometry in male military veterans. For the DHEA quantifications (Beckman Coulter), the sensitivity of the RIA is 0.06 ng/mL, the intra-assay coefficient of variation is 3.8%, and the inter-assay coefficient of variation is 8.6%. For the DHEAS quantifications (Beckman Coulter), the sensitivity of the RIA is 12.33 ng/mL, the intra-assay coefficient of variation is 5.2%, and the inter-assay coefficient of variation is 5.3% (please note, DHEAS quantifications can only be quantified indirectly by RIA following cleavage of the sulfate group). For both DHEA and DHEAS, cross-reactivity with other steroids was described by the manufacturer as being “extremely low.” For progesterone (MP Biomedical), the sensitivity is 0.05 ng/mL, the commercial intra-assay coefficient of variation is 2.3% and the inter-assay coefficient of variation is 4.9%. Cross-reactivity for the progesterone RIA was reported by the manufacturer for 20 steroids as follows: for two steroids tested, cross-reactivity was <5.5% (i.e., 20α-dihydroprogesterone = 5.41%, desoxycorticosterone 3.80%), for five additional steroids tested cross-reactivity was <1.0%, and for the remaining 13 steroids tested cross-reactivity was <0.01%. Secondary to limited serum volume availability, RIAs were not run in duplicate, and the above coefficients of variation represent commercial values reported by the manufacturer.

NS quantification of the remaining neuroactive steroids was performed by highly sensitive and specific GC/MS preceded by a high performance liquid chromatography (HPLC) purification step. Serum was homogenized in distilled water containing 2 fmol of tritiated NS of interest (New England Nuclear) and 400 pg of the deuterium-labeled NS of interest (Cambridge Isotope Laboratories). Supernatants were extracted three times with ethyl acetate and dried under nitrogen prior to HPLC, as described by Sripada et al. (2013a, b). HPLC purification was performed on an 1100 Series Agilent HPLC utilizing hexane, tetrahydrofuran, and ethanol as the mobile phase and an Alltech LiChrosorb DIOl 250 mm × 4.6 mm column. The HPLC fractions containing the NS of interest were derivatized utilizing heptafluorobutyric acid anhydride (HFBA).

Mass spectrometry for the derivatized steroids was performed in the EI mode utilizing helium as the carrier gas on an Agilent 5973 MS coupled to an Agilent 6890N GC equipped with an Agilent HP-5MS 30 m × 0.250 mm × 0.25 um capillary column. In addition to the GC retention time characteristic of each steroid, the structural identification of each NS assayed was provided by its unique mass fragmentation pattern. We utilized the single ion-monitoring (SIM) mode of the mass spectrometer to focus on the most abundant ion fragment for each steroid derivative. For NS quantification, the standard curve for the steroid of interest was prepared by combining varying known quantities of steroids (Steraloids) ranging from 1 to 3000 pg with a constant amount of deuterated internal standard. The area under the peak of the internal standard was divided by the area under the peak of the known quantity of each steroid and plotted against the quantity of each steroid to generate the standard curve. Limit of NS detection with this method is 1 pg for each NS. A subset of the samples (7%) was quantified in duplicate; intra-assay coefficients of variation were 4.3% for allopregnanolone, 1.8% for pregnenolone, 1.0% for pregnanolone, and 2.0% for androsterone.

The blood sample was obtained in the resting state with the participant seated comfortably in an ambulatory clinic setting. The participant had not eaten or smoked for at least 30 min prior to the blood draw based on the duration of study procedures that preceded the blood draw. The blood draw preceded the MRI scan for all subjects (sample-1 mean = 1.37 years SD = 1.83; sample-2 mean 1.25 years, SD = 1.80). RIA was utilized to quantify serum levels of DHEA (Beckman Coulter), progesterone (MP Biomedical), and DHEAS (Beckman Coulter), according to manufacturer directions. The time range between blood draw and MRI scan for sample-1 and sample-2 was from 1 day (0.003 years) up to 3,259 days (8.9 years). Moreover 87.4% (125/143) of participants underwent MRI scanning within 3 years of blood draw.

### Statistical Analysis

Analyses of serum neuroactive steroid levels and MRI cortical thickness were conducted in male participants for whom both an MRI scan and neuroactive steroid levels were available (*n* = 115 for allopregnanolone, pregnenolone, androsterone, pregnanolone, progesterone; *n* = 143 for DHEA and DHEAS levels). Zero-order (bivariate) Pearson correlations were computed in the initial stage between all pairings of seven neuroactive steroids and left hemisphere, right hemisphere, and bilateral global gray matter volume. The corresponding correlation coefficient (*r*) and *p*-value for each correlation was Bonferroni corrected for 21 multiple tests (7 neuroactive steroids × 3 brain measures). Local and regional neurostructural associations were investigated in the initial stage with zero-order (bivariate) Pearson correlations computed between all pairings of seven neuroactive steroid and 148 regional cortical thickness measures (1,036 comparisons). The corresponding *t*-statistic and *p*-value for each correlation was corrected for multiple testing by controlling the False Discovery Rate (FDR) as reported by Benjamini and Hochberg (1995). For comparison, the adaptive FDR procedure developed by Storey et al. (2004) and Bonferroni correction (Perneger, 1998) were also applied. In cases where a high proportion of the null hypotheses are false, as in the case of widespread effects such as cortical thinning, adaptive FDR methods can paradoxically reject more null hypotheses than using uncorrected *p*-values (Reiss et al., 2012).

The main analyses were performed with ordinary least squares (OLS) regression procedures on the findings from the initial stage that survived the Benjamini and Hochberg FDR correction. Regional cortical thickness was regressed on a set of independent variables including serum neuroactive steroid level, age, depression score, alcohol use, PTSD diagnosis, childhood trauma exposure, antidepressant medication use, and smoking status. All participants were male, and thus no adjustment for sex was necessary. Five neuroactive steroids out of the seven had significant associations with cortical regions that persisted following FDR correction (*p* < 0.05) for multiple testing based on 1,036 tests (7 neuroactive steroids × 148 brain regions). These included allopreganolone (59 regions), pregnenolone (46 regions), DHEAS (46 regions), DHEA (13 regions), and androsterone (6 regions). To avoid overfitting, predictor variables lacking associations (*p* > 0.1) were removed from the regression model and the model was re-estimated with the remaining predictors. Among the tested covariates, only age was a consistent predictor of cortical thickness for all five neuroactive steroid candidates. Based on results that frequently showed both age and neuroactive steroid level as significant predictors, we estimated a second set of models testing age as a modulating (interaction) factor in the association between neuroactive steroid levels and cortical thickness. To visualize the results of the statistical interaction between age and neuroactive steroid level, we dichotomized age to young (<40 years) and old (>40 years). An age-cutoff of 40 years was chosen based on (1) an observed significant decline at age 40 years (polynomial fit), particularly for allopreganolone, and (2) median age of the sample was 40 years. Finally, we examined whether the time elapsed between the blood draw and MRI scan differentially influenced cortical thickness based on the neuroactive steroid level by calculating the interaction of years elapsed and neuroactive steroid level.

We tested whether the correlation between neursteroid level and cortical thickness grows stronger (or weaker) when the duration between blood draw and MRI scan is short (<200 days) or when it is long (>200 days). We selected 200 days because it was the median split in the number of days between blood draw and MRI scan. A significant difference in the correlation between short and long intervals, which could be visualized as different slopes of the fitted line for short and long durations, would indicate that the elapsed time between blood draw and MRI is affecting the relationship between neuroactive steroid level and cortical thickness. The interaction was tested with the R function *cocor* (Diedenhofen and Musch, 2015).

### Vertex-Wide Analysis

Cortical thickness surface maps were generated following registration of all participants’ cortical reconstructions to the FreeSurfer common average surface. Surface maps were smoothed by use of default kernel of 10 mm FWHM. The statistical parametric analysis was performed by the built-in Qdec pipeline implemented in FreeSurfer, by use of a general linear model. Cortical thickness across the whole cortical surface was regressed on a set of independent variables including serum neuroactive steroid level, age, depression score, alcohol use, PTSD diagnosis, childhood trauma exposure, antidepressant medication use, and smoking status. All participants were male, and thus no adjustment for sex was necessary. Correction for multiple comparisons was achieved with a voxel-wise cluster-forming threshold (CFT) of *p* < 0.01 and cluster-wise threshold of *p* < 0.05, Monte Carlo corrected based on 10,000 simulations (Greve and Fischl, 2017).

## Results

### Demographic and Clinical Characteristics

Demographic and clinical information are reported in Table 1 with group mean and descriptive statistics reported separately for the subsample (*n* = 115) and full-sample (*n* = 143). Assays for pregnenolone, allopregnanolone, pregnanolone, androsterone, and progesterone were performed in only the subsample.

**TABLE 1:** Clinical and demographic features of sample.

	**Subsample**		**Full-sample**	
	**(*n* = 115)**		**(*n* = 143)**	
		
**Clinical parameter**	**Mean/**	**SD/**	**Mean/**	**SD/**
	**No.**	**Percent**	**No.**	**Percent**
Age (years)	38.60	7.14	39.64	9.78
BDI	12.08	12.56	11.4	12.28
PTSD diagnosis	55	47.83%	69	48.25%
Antidepressant use	22	19.13%	28	19.58%
TLEQ Child Trauma – 0 Categories	59	51.30%	76	53.14%
TLEQ Child Trauma – 1 Categories	21	18.26%	24	16.78%
TLEQ Child Trauma – 2+ Categories	20	17.39%	21	14.69%
Alcohol use disorders test	4.33	4.86	3.96	4.56
Current smokers	23	20.00%	29	20.28%
Former smokers	30	26.09%	38	26.57%
Non-smokers	62	53.91%	75	52.45%
Time elapsed, blood draw to scan (years)	1.25	1.80	1.37	1.83

*SD/Percent, standard deviation or percentage of sample; BDI, beck depression inventory; TLEQ, trauma life events questionnaire (Child Trauma was coded as the number of categories of trauma experienced).*

### Association of Neuroactive Steroids and Regional Cortical Thickness

We found highly significant correlations of left (L), right (R), and bilateral (B/L) hemisphere gray matter volume with allopregnanolone (L: *r* = 0.458, *p* = 1.86 × 10^–6^; R: *r* = 0.459, *p* = 1.64 × 10^–6^; B/L: *r* = 0.460, *p* = 1.62 × 10^–6^; corrected) and pregnenolone (L: *r* = 0.355, *p* = 0.001; R: *r* = 0.360, *p* = 0.001; B/L: *r* = 0.358, *p* = 0.001; corrected). The relationships of hemispheric cortical volume with the remaining neuroactive steroids were non-significant after correction for the number of neuroactive steroids tested (Table 2). The significant associations of global mean values with neuroactive steroids prompted a finer grained investigation of the association between regional cortical thickness and neuroactive steroid levels.

**TABLE 2:** Neurosteroid associations with global gray matter volume by hemisphere.

	**DHEA**		**DHEAS**		**Allopregnanolone**		**Pregnanolone**		**Androsterone**		**Progesterone**		**Pregnenolone**	
							
	***r***	***p***	***r***	***p***	***r***	***p***	***r***	***p***	***r***	***p***	***r***	***p***	***r***	***p***
Left	0.153	0.472	0.108	>0.9	0.458	1.86E−06	0.035	>0.9	0.214	0.150	0.193	0.268	0.355	0.001
Right	0.157	0.427	0.126	>0.9	0.459	1.64E−06	0.044	>0.9	0.197	0.245	0.180	0.375	0.360	0.001
B/L	0.156	0.444	0.117	>0.9	0.460	1.62E−06	0.040	>0.9	0.206	0.190	0.187	0.314	0.358	0.001

*DHEA, Dehydroepiandrosterone; DHEAS, Dehydroepiandrosterone Sulfate; B/L, bilateral.*

**FIGURE 1:**
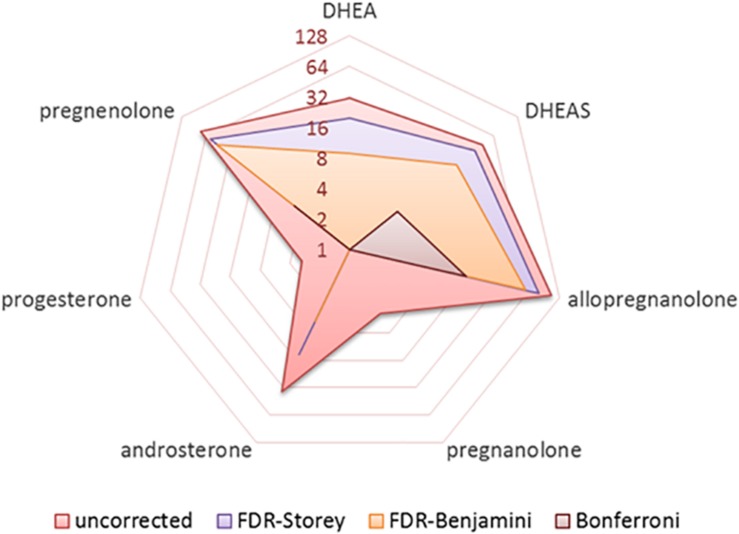
Radar plot showing the number of significant results (log base-2) obtained from bivariate correlations of seven neurosteroids with 148 cortical regions (1036 total tests) for various approaches to handling multiple comparisons including uncorrected, FDR correction of Benjamini and Hochberg, pFDR correction of Storey, and Bonferroni correction.

The number of significant results from bivariate correlations of the seven neuroactive steroids with 148 cortical regions are shown in Figure 1 using various approaches to multiple comparison testing: (1) uncorrected, (2) adaptive FDR correction of Storey (Storey et al., 2004), (3) FDR correction of Benjamini and Hochberg (1995), and (4) Bonferroni correction (Perneger, 1998). Final regression analyses were conducted on significant results identified by FDR criteria (Benjamini and Hochberg, 1995).

Five neuroactive steroids, specifically allopregnanolone, pregnenolone, DHEAS, DHEA, and androsterone, were significantly correlated with cortical thickness in multiple brain regions [*p* < 0.05; FDR-corrected (1995)]. Two neuroactive steroids, pregnanolone and progesterone, did not exhibit significant bivariate associations with cortical regions that persisted following FDR correction (1995).

#### Allopregnanolone

**FIGURE 2:**
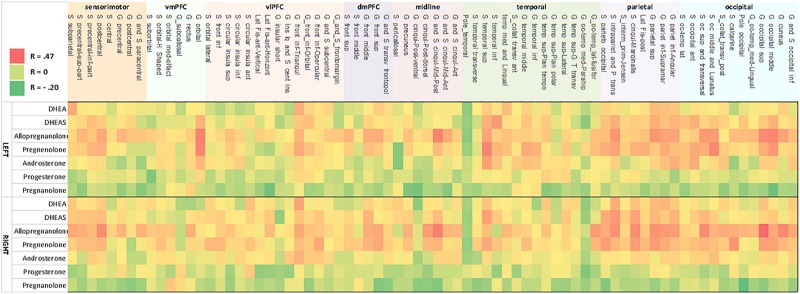
Heat map of correlation between neurosteroid level and cortical thickness. Correlation strength between neurosteroid level (*y*-axis) and cortical thickness (*x*-axis) for the left hemisphere (upper heat map) and right hemisphere (lower heat map). The pattern shows concordant relationships among DHEAS, allopregnanolone, pregnenolone, and to a lesser extent with androsterone as well as concordant results between the left and right hemisphere. The strongest correlations are present in inferior frontal cortex, temporoparietal, occipitoparietal, and occiptiotemporal association cortices. Nomenclature of cortical structure is per definitions provided by Destrieux et al. (2010).

Bivariate correlations revealed that allopregnanolone had significant associations with gray matter cortical thickness in 59 cortical regions (*p* < 0.05; FDR-corrected). Regression analysis with all predictor variables revealed that aside from neuroactive steroid level, only age contributed significantly to the model. The remaining variables, depression score, alcohol use, PTSD diagnosis, childhood trauma exposure, antidepressant medication use, and smoking status were non-significant with respect to cortical thickness (FDR-corrected). Results from a restricted model including only age and neuroactive steroid level showed age was significantly associated with all 59 regions that were identified in the bivariate correlations, and that neuroactive steroid level was significantly associated with cortical thickness in 21 regions, including: left middle posterior cingulate gyrus/sulcus, left cuneus gyrus, left middle occipital gyrus, left superior occipital gyrus, left occipito-temporal and medial lingual gyrus, left orbital gyrus, left superior parietal gyrus, left intraparietal sulcus, left occipital and middle lunatus, left occipital superior gyrus, and transversal sulcus, right middle posterior cingulate gyrus/sulcus, right cuneus, right middle occipital gyrus, right superior occipital gyrus, right occipito-temporal lateral fusiform, right occipito-temporal medial and lingual gyrus, right superior parietal gyrus, right rectus gyrus, right marginal cingulate sulcus, right transverse posterior collateral sulcus, and right postcentral sulcus (Figure 2 and Table 3).

**TABLE 3:** Allopregnanolone association with cortical thickness.

**Cortical region^†^**	**Age**	**Beta (NS)**	***p* (NS)**	***t* (NS)**	***r***	***p* (FDR)**
L_G_and_S_cingul-Mid-Ant #	2.21E−02	0.0009	0.050	1.982	0.228	0.0115
**L_G_and_S_cingul-Mid-Post #**	8.41E−04	0.0010	0.003	3.061	0.282	0.0002
L_G_and_S_occipital_inf	4.51E−02	0.0007	0.138	1.495	0.400	0.0476
**L_G_cuneus**	4.29E−01	0.0009	0.006	2.824	0.308	0.0051
L_G_front_inf-Triangul	2.89E−08	0.0002	0.599	0.527	0.255	0.0234
L_G_front_middle	1.47E−05	0.0004	0.239	1.183	0.275	0.0127
L_G_front_sup	1.32E−05	0.0004	0.225	1.220	0.278	0.0122
**L_G_occipital_middle #**	2.13E−04	0.0009	0.007	2.737	0.384	0.0002
**L_G_occipital_sup #**	3.21E−02	0.0012	0.002	3.140	0.376	0.0003
**L_G_oc-temp_med-Lingual #**	6.03E−01	0.0008	0.031	2.183	0.240	0.0351
**L_G_orbital**	6.28E−05	0.0012	0.005	2.863	0.400	0.0002
L_G_pariet_inf-Angular	2.38E−05	0.0006	0.116	1.586	0.305	0.0055
L_G_pariet_inf-Supramar	5.19E−05	0.0006	0.098	1.669	0.307	0.0051
**L_G_parietal_sup #**	6.77E−05	0.0007	0.040	2.079	0.339	0.0017
L_G_precentral	3.45E−03	0.0006	0.164	1.400	0.251	0.0258
L_Pole_occipital #	1.25E−01	0.0006	0.094	1.687	0.230	0.0467
L_S_calcarine	2.33E−03	0.0004	0.275	1.097	0.227	0.0476
L_S_front_sup	8.38E−03	0.0005	0.132	1.516	0.252	0.0256
**L_S_intrapariet_and_P_trans**	1.01E−04	0.0005	0.030	2.204	0.347	0.0013
**L_S_oc_middle_and_Lunatus**	1.39E−04	0.0007	0.049	1.987	0.327	0.0029
**L_S_oc_sup_and_transversal**	4.64E−03	0.0007	0.019	2.387	0.334	0.0022
L_S_parieto_occipital	4.68E−07	0.0001	0.707	0.376	0.227	0.0476
L_S_postcentral #	3.95E−03	0.0005	0.063	1.881	0.292	0.0095
L_S_precentral-sup-part	8.93E−04	0.0004	0.209	1.263	0.251	0.0256
L_S_temporal_sup	2.68E−07	0.0004	0.189	1.322	0.308	0.0051
R_G_and_S_cingul-Ant	2.32E−02	0.0002	0.694	0.395	0.291	0.0333
R_G_and_S_cingul-Mid-Ant #	2.62E−03	0.0009	0.071	1.825	0.262	0.0256
**R_G_and_S_cingul-Mid-Post #**	1.40E−01	0.0008	0.042	2.057	0.242	0.0002
R_G_and_S_occipital_inf	1.16E−03	0.0002	0.638	0.472	0.252	0.0097
R_G_and_S_paracentral	1.02E−01	0.0009	0.079	1.776	0.405	0.0193
R_G_cingul-Post-dorsal	1.69E−04	0.0008	0.057	1.922	0.321	0.0034
**R_G_cuneus #**	1.84E−01	0.0011	0.001	3.503	0.383	0.0002
R_G_front_inf-Triangul	1.19E−04	0.0005	0.133	1.514	0.289	0.0103
R_G_front_middle	1.41E−07	0.0003	0.384	0.875	0.275	0.0127
R_G_front_sup	6.66E−03	0.0007	0.065	1.867	0.285	0.0113
**R_G_occipital_middle #**	8.94E−02	0.0008	0.028	2.220	0.284	0.0115
**R_G_occipital_sup #**	8.61E−02	0.0018	0.000	4.544	0.471	0.0000
**R_G_oc-temp_lat-fusifor**	3.53E−01	0.0015	0.004	2.960	0.325	0.0029
**R_G_oc-temp_med-Lingual #**	3.10E−01	0.0007	0.048	2.000	0.240	0.0343
R_G_pariet_inf-Angular	3.24E−07	0.0005	0.127	1.539	0.324	0.0029
R_G_pariet_inf-Supramar #	1.30E−06	0.0006	0.090	1.708	0.331	0.0024
**R_G_parietal_sup #**	1.24E−03	0.0008	0.015	2.459	0.350	0.0012
R_G_precentral	1.29E−02	0.0007	0.109	1.614	0.256	0.0234
R_G_precuneus	4.81E−04	0.0004	0.190	1.318	0.261	0.0195
**R_G_rectus**	6.71E−01	0.0014	0.013	2.525	0.268	0.0158
R_Lat_Fis-ant-Horizont	6.61E−04	0.0005	0.281	1.084	0.238	0.0364
R_Pole_occipital #	1.03E−01	0.0006	0.065	1.867	0.250	0.0263
R_S_calcarine	1.86E−04	0.0005	0.172	1.375	0.274	0.0130
**R_S_cingul-Marginalis**	4.34E−04	0.0008	0.010	2.624	0.371	0.0004
**R_S_collat_transv_post**	7.27E−02	0.0009	0.036	2.119	0.279	0.0122
R_S_interm_prim-Jensen	9.94E−06	0.0000	0.937	–0.080	0.167	0.0476
R_S_oc-temp_lat	7.20E−05	0.0001	0.821	0.227	0.179	0.0115
R_S_oc-temp_med_and_Lingual	5.08E−04	0.0004	0.245	1.168	0.247	0.0002
R_S_orbital-H_Shaped	7.91E−03	0.0005	0.297	1.049	0.209	0.0051
**R_S_postcentral #**	1.08E−01	0.0009	0.002	3.204	0.366	0.0234
R_S_precentral-inf-part	6.44E−03	0.0004	0.188	1.323	0.237	0.0127
R_S_precentral-sup-part	1.39E−02	0.0006	0.070	1.832	0.274	0.0122
R_S_temporal_inf	3.05E−03	–0.0006	0.207	–1.269	–0.005	0.0002
R_S_temporal_sup	2.66E−08	0.0001	0.843	0.198	0.228	0.0003

*The Beta (NS), *p* (NS), and *t* (NS) correspond to the independent variable for neurosteroid level. The *r* column contains the result of the zero-order bivariate Pearson correlation between neurosteroid level and cortical thickness. The *p* column contains the FDR-corrected *p*-value associated with the correlation. Regions with significant *p*(NS) values are in bold typeface. Regions with significant associations bilaterally (left and right) are followed by “#.” ^†^The nomenclature used for cortical segmentation as well as the procedure of segmentation is provided in Destrieux et al. (2010).*

#### Pregnenolone

Bivariate correlations revealed pregnenolone had significant associations with 46 cortical regions (*p* < 0.05; FDR-corrected). Regression analysis with all predictor variables revealed that only age contributed significantly to the model (FDR-corrected). Results showed a significant association of age with cortical thickness for all 46 regions and a significant association of neuroactive steroid level with cortical thickness for eight regions, including: left middle occipital gyrus, left orbital gyrus, left occipital and middle lunatus, left superior temporal gyrus, right middle occipital gyrus, right superior occipital gyrus, right superior parietal gyrus, right calcarine sulcus (Figure 2 and Table 4). Associations between cortical thickness and neuroactive steroid levels, particularly for allopregnanolone and pregnenolone, modulated many of the same neuroanatomical regions. For instance, the results of bivariate correlations revealed that allopregnanolone and pregnenolone showed concordance in modulating 33 of the same neuroanatomical regions in the initial model utilizing all of the above predictor variables (Table 4).

**TABLE 4:** Pregnenolone association with cortical thickness.

**Cortical region^†^**	**Age^∗^**	**Beta (NS)**	***p* (NS)**	***t* (NS)**	***r***	***p* (FDR)**
L_G_and_S_cingul-Mid-Post	6.76E−05	3.88E−05	0.392	0.860	0.238	0.0364
L_G_front_inf-Triangul	5.66E−08	4.03E−05	0.375	0.890	0.283	0.0115
L_G_front_middle	5.87E−06	2.39E−05	0.591	0.538	0.227	0.0476
**L_G_occipital_middle^‡^**	1.23E−04	9.53E−05	0.031	2.186	0.345	0.0013
L_G_occipital_sup^‡^	1.04E−02	8.9E−05	0.075	1.798	0.276	0.0127
L_G_oc-temp_lat-fusifor^‡^	2.92E−01	0.0001	0.069	1.839	0.227	0.0476
**L_G_orbital^‡^**	5.60E−05	0.0001	0.008	2.693	0.389	0.0002
L_G_pariet_inf-Angular	5.63E−06	2.73E−05	0.581	0.553	0.229	0.0476
L_G_pariet_inf-Supramar	3.25E−05	5.53E−05	0.204	1.277	0.279	0.0122
L_G_temporal_inf	4.30E−02	0.0001	0.070	1.827	0.261	0.0198
L_G_temporal_middle	8.50E−03	9.49E−05	0.061	1.889	0.286	0.0113
L_Lat_Fis-post	9.00E−04	6.53E−05	0.142	1.481	0.272	0.0138
L_S_cingul-Marginalis	5.54E−05	4.63E−05	0.261	1.130	0.263	0.0188
L_S_intrapariet_and_P_trans	4.88E−05	5.08E−05	0.121	1.563	0.301	0.0068
**L_S_oc_middle_and_Lunatus^‡^**	3.44E−04	0.0001	0.007	2.756	0.384	0.0002
L_S_oc_sup_and_transversal	2.14E−03	6.52E−05	0.112	1.600	0.274	0.0128
L_S_occipital_ant	1.36E−04	9.18E−05	0.144	1.472	0.286	0.0113
L_S_parieto_occipital	1.32E−06	3.97E−05	0.325	0.989	0.275	0.0127
L_S_postcentral	2.75E−03	5.62E−05	0.135	1.507	0.264	0.0186
L_S_precentral-inf-part	1.57E−03	5.58E−05	0.174	1.370	0.257	0.0228
L_S_temporal_inf	2.95E−02	9.44E−05	0.102	1.648	0.249	0.0265
**L_S_temporal_sup**	1.51E−06	9.64E−05	0.011	2.573	0.397	0.0002
R_G_and_S_cingul-Mid-Post	2.16E−02	−7.6E−06	0.883	–0.148	0.260	0.0204
R_G_cingul-Post-dorsal	4.20E−05	4.21E−05	0.413	0.822	0.238	0.0364
R_G_cuneus^‡^	5.53E−02	7.61E−05	0.087	1.726	0.248	0.0276
R_G_front_inf-Opercular	2.46E−05	6.46E−05	0.168	1.387	0.290	0.0098
R_G_front_inf-Triangul	4.60E−05	3.44E−05	0.436	0.781	0.234	0.0416
R_G_front_middle	8.90E−08	2.31E−05	0.573	0.566	0.254	0.0238
R_G_front_sup^‡^	7.41E−03	9.43E−05	0.055	1.940	0.292	0.0095
**R_G_occipital_middle^‡^**	1.14E−01	0.000116	0.013	2.527	0.309	0.0051
**R_G_occipital_sup^‡^**	1.72E−02	0.0001	0.025	2.277	0.312	0.0046
R_G_oc-temp_lat-fusifor^‡^	2.05E−01	0.000133	0.050	1.978	0.248	0.0273
R_G_pariet_inf-Angular	3.86E−08	8.03E−06	0.846	0.195	0.228	0.0476
R_G_pariet_inf-Supramar	4.67E−07	4.12E−05	0.335	0.968	0.279	0.0122
**R_G_parietal_sup^‡^**	8.91E−04	8.78E−05	0.038	2.098	0.325	0.0029
R_G_temporal_middle	1.72E−03	5.59E−05	0.275	1.097	0.231	0.0450
R_Lat_Fis-post	1.62E−04	4.09E−05	0.332	0.974	0.241	0.0337
**R_S_calcarine**	5.69E−04	0.0001	0.024	2.286	0.343	0.0014
R_S_cingul-Marginalis^‡^	2.25E−04	7.5E−05	0.050	1.980	0.325	0.0029
R_S_front_inf	3.38E−02	6.44E−05	0.115	1.589	0.242	0.0330
R_S_oc-temp_med_Lingual	1.19E−04	3.41E−06	0.937	0.079	0.162	0.0364
R_S_postcentral	3.40E−02	6.34E−05	0.102	1.647	0.247	0.0115
R_S_precentral-inf-part	6.21E−03	5.38E−05	0.209	1.263	0.233	0.0476
R_S_temporal_inf	5.13E−02	8.08E−05	0.183	1.340	0.213	0.0013
R_S_temporal_sup	1.21E−07	3.71E−05	0.293	1.056	0.293	0.0127
R_S_temporal_transverse	3.17E−04	4.07E−05	0.631	0.481	0.191	0.0476

*The beta (NS), *p* (NS), and *t* (NS) correspond to the independent variable for neurosteroid level. The *r* column contains the result of the zero-order bivariate Pearson correlation between neurosteroid level and cortical thickness. The *p* column contains the FDR-corrected *p*-value associated with the correlation. Regions with significant *p* (NS) values are in bold typeface. Regions with significant associations bilaterally (left and right) are followed by “^‡^.” ^†^The nomenclature used for cortical segmentation as well as the procedure of segmentation is provided in Destrieux et al. (2010). ^∗^The predictors that were significant in the first iteration of the regression model and only these predictors and neurosteroid level were retained in the final model.*

#### Androsterone

Bivariate correlations revealed androsterone had significant associations (*p* < 0.05; FDR-corrected) with six cortical regions (Figure 2 and Table 5). In subsequent regression modeling, only age and androsterone retained significant associations with cortical thickness. Accordingly, the models were re-estimated in each of the six identified regions with only the latter two covariates retained. Regression analysis with all predictor variables revealed that age contributed significantly to the model (FDR-corrected). Results were similar across each of the six models: only age remained significantly associated with cortical thickness (Table 5).

**TABLE 5:** Androsterone association with cortical thickness.

**Cortical region^†^**	**Age^∗^**	**Beta**	***p* (NS)**	***t* (NS)**	***r***	***p***
		**(NS)**				**(FDR)**
L_G_orbital	2.6E−06	0.0002	0.236	1.192	0.245	0.0308
L_G_pariet_inf-Angular	7.04E−06	0.0002	0.118	1.574	0.272	0.0138
L_G_pariet_inf-Supramar	8.19E−06	0.0001	0.302	1.036	0.226	0.0482
L_S_occipital_ant	6.46E−05	0.0003	0.072	1.814	0.281	0.0118
L_S_temporal_sup	1.23E−07	0.0002	0.058	1.913	0.313	0.0046
R_G_front_inf-Triangul	4.29E−05	0.0002	0.124	1.550	0.262	0.0195

*The beta (NS), *p* (NS), and *t* (NS) correspond to the independent variable for neurosteroid level. The *r* column contains the result of the zero-order bivariate Pearson correlation between neurosteroid level and cortical thickness. The *p* column contains the FDR-corrected *p*-value associated with the correlation. ^†^The nomenclature used for cortical segmentation as well as the procedure of segmentation is provided in Destrieux et al. (2010). ^∗^The predictors that were significant in the first iteration of the regression model and only these predictors and neurosteroid level were retained in the final model.*

#### DHEA

Bivariate correlations identified significant (*p* < 0.05; FDR-corrected) associations between thickness and DHEA in 13 cortical regions (Figure 2 and Table 6). In subsequent regression modeling, only age and DHEA retained significant associations with cortical thickness. Accordingly, the models were re-estimated in each of the 13 identified regions with only the latter two covariates retained. Results from a restricted model including only age and neuroactive steroid level showed a significant association with age for all 13 regions and a significant association with cortical thickness in the triangularis portion of the left and right inferior frontal gyrus (Table 6).

**TABLE 6:** Dehydroepiandrosterone association with cortical thickness.

**Cortical region^†^**	**Age^∗^**	**Beta (NS)**	***p* (NS)**	***t* (NS)**	***r***	***p* (FDR)**
L_G_front_inf-Triangul	6.99E−07	0.0007	0.475	0.717	0.249	0.0055
L_G_pariet_inf-Supramar	4.78E−05	0.0088	0.334	0.970	0.181	0.0350
L_G_parietal_sup	8.11E−07	–0.0172	0.695	0.392	0.201	0.0243
L_S_oc-temp_med_and_Lingual	5.07E−05	–0.0053	0.983	0.021	0.209	0.0243
L_S_subparietal	2.33E−03	–0.0143	0.182	1.340	0.181	0.0049
**R_G_front_inf-Triangul**	3.08E−04	0.0379	0.018	2.393	0.280	0.0026
R_G_occipital_sup	9.47E−04	–0.0107	0.316	1.007	0.141	0.0350
R_G_pariet_inf-Supramar	1.38E−07	0.0365	0.346	0.945	0.231	0.0125
R_G_parietal_sup	1.13E−05	0.0007	0.417	0.814	0.197	0.0236
R_S_cingul-Marginalis	4.78E−04	–0.0201	0.491	0.690	0.224	0.0350
R_S_circular_insula_ant	6.39E−02	0.0841	0.216	1.244	0.223	0.0350
R_S_intrapariet_and_P_trans	3.06E−06	0.0003	0.435	0.783	0.240	0.0051
R_S_temporal_sup	4.64E−08	0.0113	0.717	0.363	0.174	0.0350

*The *t* (NS), *p* (NS), and beta (NS) correspond to the independent variable for neurosteroid level. The r column contains the result of the zero-order bivariate Pearson correlation between neurosteroid level and cortical thickness. The *p* column contains the FDR-corrected *p*-value associated with the correlation. Regions with significant *p*(NS) values are in bold typeface. ^∗^indicates the predictors that were significant in the first iteration of the regression model and only these predictors and neurosteroid level were retained in the final model. ^†^The nomenclature used for cortical segmentation as well as the procedure of segmentation is provided in Destrieux et al. (2010).*

#### DHEAS

Bivariate correlations revealed DHEAS had significant associations (*p* < 0.05; FDR-corrected) with 46 cortical regions (Figure 2 and Table 7). Regression analysis with all predictor variables revealed that only age contributed significantly to the model. Results from a reduced model including only age and neuroactive steroid level showed a significant association with age for all 46 regions and a significant association with cortical thickness in the left inferior temporal sulcus (Table 7).

**TABLE 7:** Dehydroepiandrosterone sulfate association with cortical thickness.

**Cortical region^†^**	**Age^∗^**	**Beta (NS)**	***p* (NS)**	***t* (NS)**	***r***	***p* (FDR)**
L_G_front_inf-Orbital	3.56E−05	6.45E−05	0.714	0.368	0.243	0.0148
L_G_front_inf-Triangul	9.09E−09	−7E-06	0.312	–1.015	0.220	0.0256
L_G_occipital_middle	1.73E−05	7.31E−05	0.535	0.622	0.156	0.0040
L_G_occipital_sup	1.29E−03	1.86E−05	0.623	0.493	0.110	0.0260
L_G_orbital	9.59E−04	0.0001	0.266	1.117	0.262	0.0036
L_G_pariet_inf-Angular	4.15E−07	3.04E−05	0.710	0.373	0.133	0.0043
L_G_pariet_inf-Supramar	2.27E−06	9.18E−06	0.882	0.149	0.192	0.0171
L_G_parietal_sup	4.87E−07	2.11E−05	0.454	0.751	0.220	0.0031
L_G_temp_sup-Plan_tempo	3.09E−07	3.79E−05	0.854	0.185	0.122	0.0290
L_G_temporal_middle	8.24E−05	2.2E−05	0.695	0.392	0.191	0.0212
L_Pole_occipital	2.06E−02	3.96E−05	0.121	1.558	0.134	0.0223
L_S_cingul-Marginalis	4.47E−05	5.23E−05	0.840	–0.203	0.183	0.0340
L_S_intrapariet_and_P_trans	2.68E−06	1.5E−05	0.296	1.050	0.198	0.0033
L_S_oc_middle_and_Lunatus	1.83E−04	5.14E−05	0.554	0.594	0.121	0.0216
L_S_occipital_ant	7.98E−06	0.0001	0.178	1.352	0.172	0.0031
L_S_oc-temp_lat	2.49E−05	7.52E−05	0.475	0.717	0.201	0.0135
L_S_parieto_occipital	4.34E−09	4.41E−05	0.896	–0.131	0.202	0.0187
L_S_postcentral	2.38E−04	−1.1E−05	0.737	0.336	0.142	0.0344
L_S_precentral-inf-part	1.00E−04	−2.4E−06	0.390	0.863	0.217	0.0135
**L_S_temporal_inf**	5.40E−03	3.08E−05	0.003	3.047	0.279	0.0002
L_S_temporal_sup	8.13E−10	1.79E−05	0.722	0.357	0.245	0.0033
R_G_and_S_cingul-Mid-Post	1.93E−04	1.91E−05	0.483	0.704	0.258	0.0096
R_G_front_inf-Opercular	3.76E−05	5.45E−05	0.902	0.124	0.235	0.0235
R_G_front_inf-Triangul	1.19E−05	2.21E−06	0.787	0.271	0.207	0.0196
R_G_front_middle	1.19E−06	1.87E−06	0.617	0.501	0.204	0.0043
R_G_occipital_middle	3.79E−02	1.51E−05	0.359	0.921	0.134	0.0245
R_G_pariet_inf-Angular	8.10E−10	−2.2E−05	0.908	–0.116	0.167	0.0043
R_G_pariet_inf-Supramar	5.60E−10	−3E-06	0.355	–0.929	0.196	0.0328
R_G_parietal_sup	3.39E−06	−1.5E−05	0.567	0.574	0.186	0.0064
R_G_precuneus	1.70E−04	5.73E−07	0.918	0.104	0.159	0.0458
R_G_temp_sup-Lateral	7.91E−05	4.73E−05	0.643	0.464	0.189	0.0243
R_Lat_Fis-ant-Horizont	2.54E−03	0.00012	0.718	0.362	0.213	0.0245
R_S_cingul-Marginalis	1.27E−03	3.8E−05	0.102	1.648	0.332	0.0007
R_S_front_inf	8.52E−03	5.21E−05	0.500	0.676	0.139	0.0368
R_S_interm_prim-Jensen	3.98E−04	6.09E−05	0.730	0.346	0.159	0.0281
R_S_intrapariet_and_P_trans	5.37E−06	−4.2E−06	0.168	1.386	0.297	0.0002
R_S_oc_middle_and_Lunatus	1.97E−01	2.22E−05	0.242	1.174	0.181	0.0335
R_S_oc_sup_and_transversal	1.44E−02	5.05E−05	0.125	1.545	0.215	0.0080
R_S_occipital_ant	3.10E−03	5.08E−05	0.791	0.266	0.215	0.0335
R_S_parieto_occipital	4.04E−04	7.02E−05	0.209	1.262	0.294	0.0028
R_S_postcentral	1.87E−02	5.17E−05	0.169	1.383	0.190	0.0135
R_S_precentral-inf-part	7.80E−04	2.46E−05	0.408	0.830	0.164	0.0199
R_S_precentral-sup-part	5.84E−04	1.45E−05	0.369	0.902	0.141	0.0208
R_S_subparietal	6.67E−04	−5.6E−06	0.655	0.448	0.217	0.0245
R_S_temporal_inf	3.99E−02	9.59E−05	0.233	1.197	0.139	0.0245
R_S_temporal_sup	1.03E−07	4.43E−05	0.310	1.019	0.306	0.0007

*The beta (NS), *p* (NS), and *t* (NS) correspond to the independent variable for neurosteroid level. The *r* column contains the result of the zero-order bivariate Pearson correlation between neurosteroid level and cortical thickness. The *p* column contains the FDR-corrected *p*-value associated with the correlation. Regions with significant *p* (NS) values are in bold typeface. ^†^The nomenclature used for cortical segmentation as well as the procedure of segmentation is provided in Destrieux et al. (2010). ^∗^The predictors that were significant in the first iteration of the regression model and only these predictors and neurosteroid level were retained in the final model.*

### Interaction of Age and Neuroactive Steroids With Regional Cortical Thickness

**FIGURE 3:**
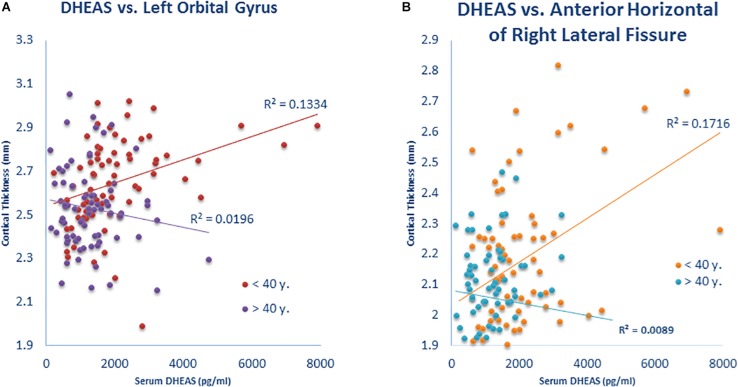
Interaction of neurosteroid level × age is associated with cortical thickness. **(A)** Participants under 40 years showed a positive correlation (*r* = 0.365) of DHEAS with cortical thickness in the left orbital gyrus but the correlation was not present (*r* = –0.140) in participants over 40 years (*Z* = 3.08; *p* = 0.002). **(B)** Participants under 40 years showed a positive correlation (*r* = 0.414) of DHEAS with cortical thickness in the anterior horizontal portion of the right lateral fissure but the correlation was not present (*r* = –0.094) in participants over 40 years (*Z* = 3.14; *p* = 0.002).

The association of cortical thickness with the interaction of neuroactive steroid level × age revealed significant results for DHEAS with left orbital gyrus (*p* = 0.04) (Figure 3A), DHEAS with the anterior horizontal part of the right lateral fissure (*p* = 0.02) (Figure 3B). This finding of positive associations between cortical thickness and DHEA and DHEAS levels in younger participants only (i.e., those younger than 40 years of age) was restricted to these two neuroactive steroids and to only two brain regions, and may thus potentially reflect Type 1 error in this exploratory analysis.

### Association of Clinical Variables With Neuroactive Steroid Levels

There were significant negative correlations of age with DHEA (*r* = −0.536; *p* = 1.5 × 10^–8^; FDR-corrected), DHEAS (*r* = −0.446; *p* = 9.4 × 10^–6^; FDR-corrected), allopregnanolone (*r* = −0.403; *p* = 7.7 × 10^–5^; FDR-corrected), androsterone (*r* = −0.323; *p* = 3.5 × 10^–3^; FDR-corrected), and pregnenolone (*r* = −0.407; *p* = 7.7 × 10^–5^; FDR-corrected). Age was not correlated with pregnanolone or with progesterone. All the associations between the remaining clinical variables (depression score, PTSD diagnosis, childhood trauma, alcohol use, medication use, smoking status) and each of the neuroactive steroids were non-significant (FDR-corrected).

### Variability in Neuroactive Steroid Levels

We quantified variability or dispersion within our sample with the coefficient of variation (CV = standard deviation/mean). The coefficient of variation was similar for all the neuroactive steroids in the current sample, except for progesterone, which had higher values (Table 8). However, the results reported in the present manuscript had no significant associations of progesterone with cortical thickness. These results were consistent with the coefficient of variation in a superset of males (*n* = 380) who had neuroactive steroid measures but lacked MRI scans (Table 8).

**TABLE 8:** Coefficient of variability for neurosteroids in multiple samples.

	**DHEA**	**DHEAS**	**Allopregnanolone**	**Pregnanolone**	**Androsterone**	**Progesterone**	**Pregnenolone**
Present MRI sample	0.418	0.630	0.610	0.534	0.538	1.624	0.594
Superset of men (*n* = 380)	0.441	0.661	0.653	0.526	0.493	1.251	0.663

### Stability of Neuroactive Steroids Over Time

**FIGURE 4:**
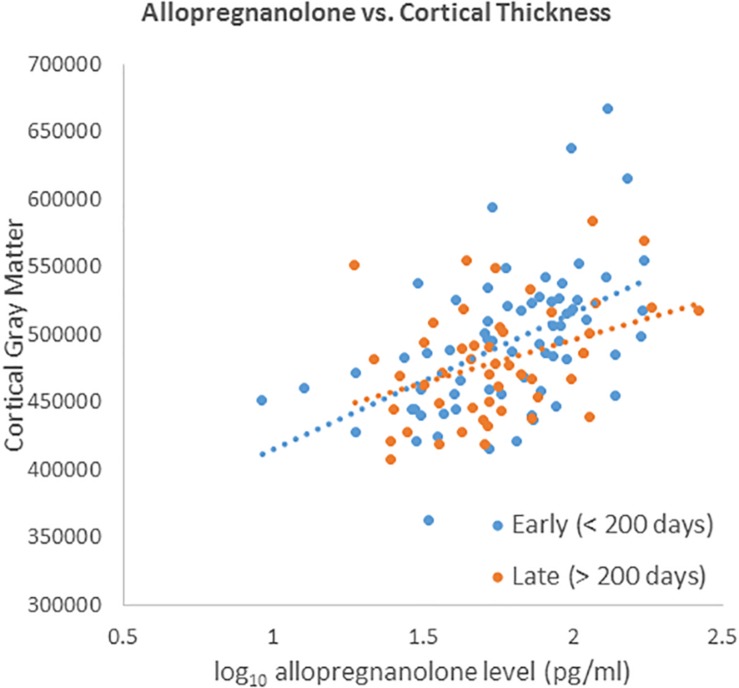
Scatter plot of log10 transformed allopregnanolone levels (*x*-axis) against cortical thickness (*y*-axis) separately for the early (blue dots/line) and late subgroups (red dots/line). Fisher’s *r*-to-*z* showed no significant difference between early (<200) and late (>200) correlations (*Z* = 0.901, *p* = 0.367).

**FIGURE 5:**
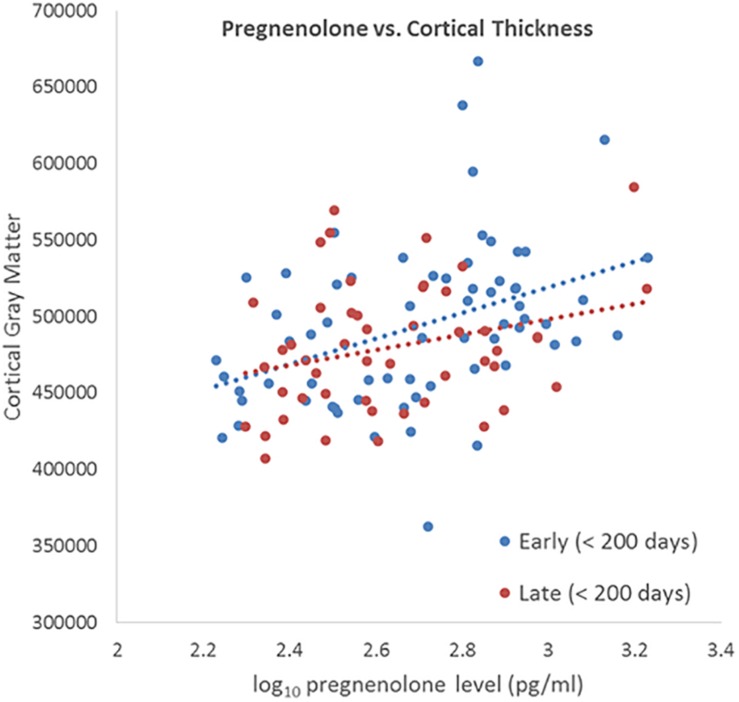
Scatter plot of log10 transformed pregnenolone levels (*x*-axis) against cortical thickness (*y*-axis) separately for the early (blue dots/line) and late subgroups (red dots/line). Fisher’s *r*-to-*z* showed no significant difference between early (<200) and late (>200) correlations (Z = 0.673, *p* = 0.501).

We did not detect a significant differential effect of time between blood draw and MRI scan on the association between neuroactive steroid levels and cortical thickness that was determined by calculating the interaction of years elapsed and neuroactive steroid level. We further tested for the stability of neuroactive steroid levels over time given the time elapsed between blood draw and MRI scan (sample-1 mean = 1.37 years, SD = 1.83 years; sample-2 mean 1.25 years, SD = 1.80 years). The sample was binned by median split of the time between blood draw and MRI scan. Half the subjects received *early* scans, within 0.548 years (200 days), and half who received *late* scans, after 0.548 years. We plotted the log base-10 transformed neuroactive steroid levels against cortical thickness separately for the early and late subgroups. The scatter plots show the correlation between both allopregnanolone (Figure 4) and pregnenolone (Figure 5). Mean cortical thickness was not significantly affected by the elapsed time between blood draw and MRI scan (allopregnanolone, *p* = 0.367; pregnenolone, *p* = 0.501). Similarly, the other five neuroactive steroids lacked a relationship with cortical thickness that was significantly influenced by time.

The correlation between neuroactive steroids levels across participants is provided as a cross-correlation (Table 9). As expected, specific neuroactive steroids were highly correlated with each other; allopregnanolone and pregnenolone were the most highly correlated (*r* = 0.494; *p* = 3.0 × 10 ^–8^), as well as pregnenolone and DHEA (*r* = 0.492; *p* = 4.1 × 10^–10^).

**TABLE 9:** Cross-correlation table of neurosteroid levels.

	**DHEA**		**DHEAS**		**Allopregnanolone**		**Pregnanolone**		**Androsterone**		**Progesterone**		**Pregnenolone**	
							
	***r***	***p***	***r***	***p***	***r***	***p***	***r***	***p***	***r***	***p***	***r***	***p***	***r***	***p***
DHEA	1.000	1.00	0.463	5.50E−09	0.332	5.05E−05	0.305	0.0002	0.464	5.25E−09	0.226	0.007	0.492	4.10E−10
DHEAS	0.463	5.50E−09	1.000	1.00	0.276	0.0008	0.086	0.305	0.367	6.47E−06	0.093	0.269	0.443	2.98E−08
Allopregnanolone	0.332	5.05E−05	0.276	0.0008	1.000	1.00	0.079	0.352	0.442	3.23E−08	–0.001	0.992	0.494	3.58E−10
Pregnanolone	0.305	0.0002	0.086	0.305	0.079	0.352	1.000	1.00	0.189	0.024	0.133	0.114	0.131	0.120
Androsterone	0.464	5.25E−09	0.367	6.47E−06	0.442	3.23E−08	0.189	0.024	1.000	1.00	0.058	0.495	0.453	1.27E−08
Progesterone	0.226	0.007	0.093	0.269	–0.001	0.992	0.133	0.114	0.058	0.495	1.000	1.00	0.194	0.020
Pregnenolone	0.492	4.10E−10	0.443	2.98E−08	0.494	3.58E−10	0.131	0.120	0.453	1.27E−08	0.194	0.020	1.000	1.00

*DHEA, Dehydroepiandrosterone; DHEAS, Dehydroepiandrosterone Sulfate.*

### Vertex-Wide Cortical Thickness Association With Neuroactive Steroids

**FIGURE 6:**
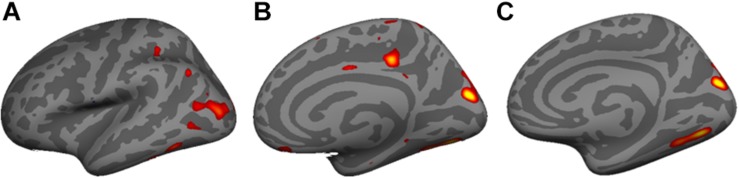
Vertex-wide cortical thickness analysis showed an association with **(A)** pregnenolone in the left lateral occipto-temporal cortex, and **(B)** with allopregnanolone in the right medial occipital cortex and **(C)** right inferior medial temporo-occipital cortex (Cluster Forming Threshold (CFT) *p* < 0.01; corrected).

Vertex-wide cortical thickness analysis showed an association with pregnenolone in the left lateral occipto-temporal cortex (Figure 6A), and with allopregnanolone in the right medial occipital cortex (Figure 6B) and right inferior medial temporo-occipital cortex (Figure 6C) (CFT *p* < 0.01; corrected).

## Discussion

Neuroactive steroid levels of allopregnanolone and pregnenolone in the peripheral circulation were positively associated with gray matter cortical thickness in multiple brain regions after FDR correction, primarily in cingulate, parietal and occipital association cortices of the brain. The effects of allopregnanolone and pregnenolone showed a similar pattern of associations with cortical thickness across many of the same brain regions. The relatively widespread nature of the FDR-corrected results suggest that the association between neuroactive steroids and cortical thickness may be more global than we report, such that brain regions with negative findings might be explained by inadequate statistical power and the influence of other modulators of cortical thickness that were not included in our model. Nevertheless, the present results are preliminary and will therefore require confirmation as elaborated below. Among the covariates we considered in the initial statistical model, only age significantly impacted the association between neuroactive steroid level and cortical thickness. Given the lack of significant covariate effects, and to avoid overfitting the model, only age was utilized as a covariate in the final set of analyses. Other covariates, while relevant to our military veteran sample (depression, past alcohol abuse, smoking, PTSD, combat exposure, and childhood trauma), were omitted. Results of the moderator analysis show that correlations between age and cortical thickness were largely independent of the association between neuroactive steroid levels and cortical thickness (except for DHEA and DHEAS in specific brain regions). We found that DHEA and DHEAS possess a strong positive correlation with specific cortical regions, such as the circular sulcus of the insula, lateral fissure, and orbital gyrus in young adults, but that this relationship is absent in relatively old adults. To our knowledge, our study is the first to describe an association between cortical thickness and neuroactive steroids in adults.

Cortical thickness quantification obtained from MRI can be influenced by a number of factors. Histology suggests cortical thinning is unlikely to originate from neuronal death, as careful post-mortem studies have found relatively comparable neuronal counts between older and younger subjects, a finding that is supported in non-human primates. Rather, cellular shrinkage and reduction in dendritic arborization are more likely to account for cortical thinning (Morrison and Hof, 1997). Developmental cortical gray-matter thinning is thought to result from both synaptic pruning and myelination (Sowell et al., 2001, 2004; O’donnell et al., 2005; Dosenbach et al., 2010). Over the course of childhood, white-matter volume expands via myelin proliferation and replaces gray matter (Sowell et al., 2004; #12189). This process results in smaller estimates of cortical gray-matter thickness (Kharitonova et al., 2013). Therefore, cortical thinning can be a consequence of increased myelination that may occur well into adulthood. For instance, myelination of a key relay zone in the hippocampal formation occurs in the human brain during childhood, adolescence, and adulthood (Benes et al., 1994). Alternatively, increase in cortical thickness accompanies learning, which correlates histologically with cellular events in gray matter that may be detected by MRI such as axon sprouting, dendritic branching and synaptogenesis, neurogenesis, changes in glial number and morphology, and angiogenesis (Zatorre et al., 2012). Given the regenerative, neuroprotective, and anti-inflammatory actions of neuroactive steroids, among other pleiotropic effects, the possible mechanistic processes of neuroactive steroids’ modulatory effects on cortical thickness are multi-faceted. Allopregnanolone is a positive allosteric modulator of both extrasynaptic and synaptic GABA_A_ receptors (potentiating GABA_A_ responses more potently than benzodiazepines or barbiturates) (Morrow et al., 1987, 1990; Lambert et al., 2009; Carver and Reddy, 2013; Reddy, 2018), which is another candidate mechanism that explains the actions of neuroactive steroids as modulators of cortical thickness (Belelli and Lambert, 2005), as well as their therapeutic potential (Irwin et al., 2014; Vallée, 2016). The neuroactive steroids allopregnanolone and pregnenolone seemed to particularly impact cortical thickness. These neuroactive steroids are not necessarily more abundant in brain (based on recent human post-mortem brain tissue investigations), and it is unclear whether they are more or less efficiently transported in the brain. While allopregnanolone and pregnenolone positively impact myelination/synaptogenesis, less is known about other neuroactive steroids such as androsterone and pregnanolone.

Although enhanced neurogenesis and other neuroactive steroid actions represent plausible mechanisms, non-neuronal factors such as changes to vasculature, which accounts for 5% of gray matter, and glial cells, which outnumber neurons 6:1, could also contribute to enhanced cortical thickness (Zatorre et al., 2012). However, because our findings are correlational and not causal, an alternative or parallel explanation is that participants who possess greater cortical thickness have more cortical gray matter volume that is capable of synthesizing neuroactive steroids (i.e., an increased capacity for synthesis of neuroactive steroids within cortical gray matter could be reflected in increased neuroactive steroid levels in the periphery). While possible, this scenario requires that neuroactive steroids synthesized in the brain make their way into the peripheral circulation, where our quantifications were obtained. Given the lipophilicity of many neuroactive steroids and their ability to cross the blood-brain-barrier (BBB) in their unsulfated forms, determining the compartmentalization and potential movements of these molecules in brain and blood has been very challenging. To date, the evidence from multiple laboratories shows that peripheral neuroactive steroids cross the BBB and subsequently cause elevations in neuroactive steroid levels in brain (Wang et al., 1997; Marx et al., 2003, 2006a). To our knowledge, there is no evidence at this time that the converse occurs, but this possibility appears tenable. It is also possible that other factors may independently impact neuroactive steroid levels and cortical thickness, such as the expression of genes regulating cellular proliferation in the brain, adrenals, and/or other regions.

Findings of relatively widespread positive correlations between cortical thickness and neuroactive steroids warrants further investigation into whether there is a possible causal relationship between neuroactive steroid levels and cortical thickness. If confirmed, further research could be pursued to assess neuroactive steroids as therapeutic agents for psychiatric and neurological conditions that are affected by cortical thinning. Reductions in neuroactive steroid molecules have been reported in multiple CNS disorders. If a contributory link between low neuroactive steroids and CNS disorder is established, amelioration of neuroactive steroid deficits through exogenous supplementation could be clinically efficacious (Irwin et al., 2014; Vallée, 2016), as supported by evidence in schizophrenia (Marx et al., 2011, 2014), bipolar disorder (Osuji et al., 2010; Brown et al., 2014), severe postpartum depression (Kanes et al., 2016), super-refractory status epilepticus (Reddy and Kuruba, 2013; Broomall et al., 2014), and TBI (Marx et al., 2016), which are associated with cortical thinning (Shaw et al., 2006; Dickerson et al., 2009; Rimol et al., 2010; Hanford et al., 2016; van Erp et al., 2016). Investigation of neuroactive steroids in CNS conditions is supported by previous neuroimaging investigations involving one-time neuroactive steroid administration and fMRI paradigms (pregnenolone 400 mg or DHEA 400 mg). We previously showed that peripheral serum allopregnanolone levels were associated with enhanced activation of emotion regulation circuits following pregnenolone administration (Sripada et al., 2013b). DHEA also modulated resting-state amygdala connectivity (Sripada et al., 2014), and DHEA administration-enhanced emotion regulation circuits and modulated memory for emotional stimuli (Sripada et al., 2013a). A causal relationship between neuroactive steroid supplementation and enhanced cortical thickness will be important to establish in future investigations.

We hypothesized that neuroactive steroids may exert stronger influence on cortical thickness for young adults but this influence might be weak in older adults, but this hypothesis was not confirmed except for the relationship of DHEAS level with thickness of the left orbital gyrus, and DHEAS levels with the anterior horizontal portion of the right lateral fissure (Figure 3). Our hypothesis was based on the declining response of the aging brain to a variety of neuromodulators. This age by neuroactive steroid interaction is also relevant to the potential for neuroactive steroids as therapeutic agents in older adults who are particularly affected by deleterious effects of cortical thinning (Morrison and Hof, 1997). The lack of an age by neuroactive steroid interaction on cortical thickness, combined with lower neuroactive steroid levels in older participants, raises the possibility that neuroactive steroids could be attractive therapeutic agents in elderly individuals who experience cortical thinning and associated cognitive decline. This proposition awaits future confirmation that neuroactive steroids effectively stimulate/preserve cortical gray matter.

### Limitations

Important caveats and limitations of this study deserve some careful discussion. The current sample of United States military veterans who served after September 11, 2001 has unique health concerns and exposures associated with deployment to Iraq and Afghanistan, which may limit the ability to generalize these results to other populations despite our efforts to statistically control for relevant variables. Confirmation of our findings in a larger independent cohort that is more representative would address this limitation. Although smoking, trauma history, depression, PTSD, and variables other than age did not appear to impact the association between neuroactive steroid levels and cortical thickness, it is possible that a larger sample may be necessary to discern these effects. Because only males participated in the current study, it is unclear whether the current findings generalize to women. We quantified peripheral (serum) neuroactive steroid levels rather than brain neuroactive steroid levels, and the precise relationships between serum and brain neuroactive steroid levels remains to be definitively elucidated. However, evidence to date suggests correlated levels between serum, CSF, and cortical regions (Marx et al., 2006a; Naylor et al., 2008; Kancheva et al., 2011). The significant time lag between obtaining blood for neurosteroid assays and the MRI scan acquisition used for ascertaining cortical thickness is a concern. However, we showed that results were consistent in a subsample who received an MRI scan within 200 days of the blood draw. Furthermore we did not detect a significant association between neuroactive steroid levels and cortical thickness that was modulated by the time elapsed between the blood draw and the MRI scan. While neuroactive steroid levels in the brain may be regionally specific, very few human studies have been conducted to date. Recent clinical and preclinical findings lend credence to an important role for allopregnanolone and pregnenolone relative to other neuroactive steroids in the modulation of brain structure and function. The current study is also cross-sectional, and thus changes in brain morphology (and potential correlations with neuroactive steroids) could not be assessed longitudinally within an individual in this pilot effort. Future studies will be required to understand these regional differences and their neurobiological underpinnings.

## Conclusion

Neuroactive steroid levels in the periphery are positively correlated with cortical thickness derived from high-resolution structural MRI in multiple brain regions. Given that neuroactive steroids exhibit pleiotropic actions that include the enhancement of neurogenesis, neuroprotection, neurotrophic properties, and anti-inflammatory effects (among others), there are likely to be multiple mechanisms that contribute to the putative modulatory actions of neuroactive steroids on cortical thickness. If our findings are confirmed in a larger and prospectively designed study, neuroactive steroid quantification and MRI assessment of cortical thickness are potential biomarkers for illness identification, therapeutic response prediction following intervention, and defining biomarker signatures of risk and resilience. Thus, our findings may stimulate new hypotheses and future avenues for investigation in this promising area.

## Data Availability Statement

Neurosteroid data is not publicly available since it is protected VA data. For access to neuroimaging data, go to https://openneuro.org/, click public database, and then search for Serum Grey Matter Cortical Thickness.

## Ethics Statement

The study has been approved by the Institutional Review Board at Duke University Medical Center and the Durham VA Medical Center. The research has been performed in accordance with the ethical standards as laid down in the 1964 Declaration of Helsinki and its later amendments or comparable ethical standards. Informed consent was obtained from all individual participants included in the study.

## Author Contributions

RM: writing the manuscript, data analysis, and final approval of the manuscript. SD and CS: data collection and final approval of the manuscript. CH and DS: data analysis and final approval of the manuscript. JN and SS: study design and final approval of the manuscript. JK: database management and final approval of the manuscript. LS and GP: neurosteroid assays and final approval of the manuscript. HW: biostatistics and final approval of the manuscript. CM: obtaining funding, writing of the manuscript, study design, and final approval of the manuscript.

## Conflict of Interest

CM is a co-applicant on pending patent applications focusing on the use of neuroactive steroids and derivatives in CNS disorders. No patents issued. No licensing in place. VA-208 waiver in place. The remaining authors declare that the research was conducted in the absence of any commercial or financial relationships that could be construed as a potential conflict of interest.

## Footnotes

1 http://surfer.nmr.mgh.harvard.edu/
